# Statistical Analysis of Complex Problem-Solving Process Data: An Event History Analysis Approach

**DOI:** 10.3389/fpsyg.2019.00486

**Published:** 2019-03-18

**Authors:** Yunxiao Chen, Xiaoou Li, Jingchen Liu, Zhiliang Ying

**Affiliations:** ^1^Department of Statistics, London School of Economics and Political Science, London, United Kingdom; ^2^School of Statistics, University of Minnesota, Minneapolis, MN, United States; ^3^Department of Statistics, Columbia University, New York, NY, United States

**Keywords:** process data, complex problem solving, PISA data, response time, event history analysis

## Abstract

Complex problem-solving (CPS) ability has been recognized as a central 21st century skill. Individuals' processes of solving crucial complex problems may contain substantial information about their CPS ability. In this paper, we consider the prediction of duration and final outcome (i.e., success/failure) of solving a complex problem during task completion process, by making use of process data recorded in computer log files. Solving this problem may help answer questions like “how much information about an individual's CPS ability is contained in the process data?,” “what CPS patterns will yield a higher chance of success?,” and “what CPS patterns predict the remaining time for task completion?” We propose an event history analysis model for this prediction problem. The trained prediction model may provide us a better understanding of individuals' problem-solving patterns, which may eventually lead to a good design of automated interventions (e.g., providing hints) for the training of CPS ability. A real data example from the 2012 Programme for International Student Assessment (PISA) is provided for illustration.

## 1. Introduction

Complex problem-solving (CPS) ability has been recognized as a central 21st century skill of high importance for several outcomes including academic achievement (Wüstenberg et al., 2012) and workplace performance (Danner et al., 2011). It encompasses a set of higher-order thinking skills that require strategic planning, carrying out multi-step sequences of actions, reacting to a dynamically changing system, testing hypotheses, and, if necessary, adaptively coming up with new hypotheses. Thus, there is almost no doubt that an individual's problem-solving process data contain substantial amount of information about his/her CPS ability and thus are worth analyzing. Meaningful information extracted from CPS process data may lead to better understanding, measurement, and even training of individuals' CPS ability.

Problem-solving process data typically have a more complex structure than that of panel data which are traditionally more commonly encountered in statistics. Specifically, individuals may take different strategies toward solving the same problem. Even for individuals who take the same strategy, their actions and time-stamps of the actions may be very different. Due to such heterogeneity and complexity, classical regression and multivariate data analysis methods cannot be straightforwardly applied to CPS process data.

Possibly due to the lack of suitable analytic tools, research on CPS process data is limited. Among the existing works, none took a prediction perspective. Specifically, Greiff et al. (2015) presented a case study, showcasing the strong association between a specific strategic behavior (identified by expert knowledge) in a CPS task from the 2012 Programme for International Student Assessment (PISA) and performance both in this specific task and in the overall PISA problem-solving score. He and von Davier (2015, 2016) proposed an N-gram method from natural language processing for analyzing problem-solving items in technology-rich environments, focusing on identifying feature sequences that are important to task completion. Vista et al. (2017) developed methods for the visualization and exploratory analysis of students' behavioral pathways, aiming to detect action sequences that are potentially relevant for establishing particular paths as meaningful markers of complex behaviors. Halpin and De Boeck (2013) and Halpin et al. (2017) adopted a Hawkes process approach to analyzing collaborative problem-solving items, focusing on the psychological measurement of collaboration. Xu et al. (2018) proposed a latent class model that analyzes CPS patterns by classifying individuals into latent classes based on their problem-solving processes.

In this paper, we propose to analyze CPS process data from a prediction perspective. As suggested in Yarkoni and Westfall (2017), an increased focus on prediction can ultimately lead us to greater understanding of human behavior. Specifically, we consider the simultaneous prediction of the duration and the final outcome (i.e., success/failure) of solving a complex problem based on CPS process data. Instead of a single prediction, we hope to predict at any time during the problem-solving process. Such a data-driven prediction model may bring us insights about individuals' CPS behavioral patterns. First, features that contribute most to the prediction may correspond to important strategic behaviors that are key to succeeding in a task. In this sense, the proposed method can be used as an exploratory data analysis tool for extracting important features from process data. Second, the prediction accuracy may also serve as a measure of the strength of the signal contained in process data that reflects one's CPS ability, which reflects the reliability of CPS tasks from a prediction perspective. Third, for low stake assessments, the predicted chance of success may be used to give partial credits when scoring task takers. Fourth, speed is another important dimension of complex problem solving that is closely associated with the final outcome of task completion (MacKay, 1982). The prediction of the duration throughout the problem-solving process may provide us insights on the relationship between the CPS behavioral patterns and the CPS speed. Finally, the prediction model also enables us to design suitable interventions during their problem-solving processes. For example, a hint may be provided when a student is predicted having a high chance to fail after sufficient efforts.

More precisely, we model the conditional distribution of duration time and final outcome given the event history up to any time point. This model can be viewed as a special event history analysis model, a general statistical framework for analyzing the expected duration of time until one or more events happen (see e.g., Allison, 2014). The proposed model can be regarded as an extension to the classical regression approach. The major difference is that the current model is specified over a continuous-time domain. It consists of a family of conditional models indexed by time, while the classical regression approach does not deal with continuous-time information. As a result, the proposed model supports prediction at any time during one's problem-solving process, while the classical regression approach does not. The proposed model is also related to, but substantially different from response time models (e.g., van der Linden, 2007) which have received much attention in psychometrics in recent years. Specifically, response time models model the joint distribution of response time and responses to test items, while the proposed model focuses on the conditional distribution of CPS duration and final outcome given the event history.

Although the proposed method learns regression-type models from data, it is worth emphasizing that we do not try to make statistical inference, such as testing whether a specific regression coefficient is significantly different from zero. Rather, the selection and interpretation of the model are mainly justified from a prediction perspective. This is because statistical inference tends to draw strong conclusions based on strong assumptions on the data generation mechanism. Due to the complexity of CPS process data, a statistical model may be severely misspecified, making valid statistical inference a big challenge. On the other hand, the prediction framework requires less assumptions and thus is more suitable for exploratory analysis. More precisely, the prediction framework admits the discrepancy between the underlying complex data generation mechanism and the prediction model (Yarkoni and Westfall, 2017). A prediction model aims at achieving a balance between the bias due to this discrepancy and the variance due to a limited sample size. As a price, findings from the predictive framework are preliminary and only suggest hypotheses for future confirmatory studies.

The rest of the paper is organized as follows. In Section 2, we describe the structure of complex problem-solving process data and then motivate our research questions, using a CPS item from PISA 2012 as an example. In Section 3, we formulate the research questions under a statistical framework, propose a model, and then provide details of estimation and prediction. The introduced model is illustrated through an application to an example item from PISA 2012 in Section 4. We discuss limitations and future directions in Section 5.

## 2. Complex Problem-Solving Process Data

### 2.1. A Motivating Example

We use a specific CPS item, *CLIMATE CONTROL* (CC)^1^, to demonstrate the data structure and to motivate our research questions. It is part of a CPS unit in PISA 2012 that was designed under the “MicroDYN” framework (Greiff et al., 2012; Wüstenberg et al., 2012), a framework for the development of small dynamic systems of causal relationships for assessing CPS.

**Figure 1:**
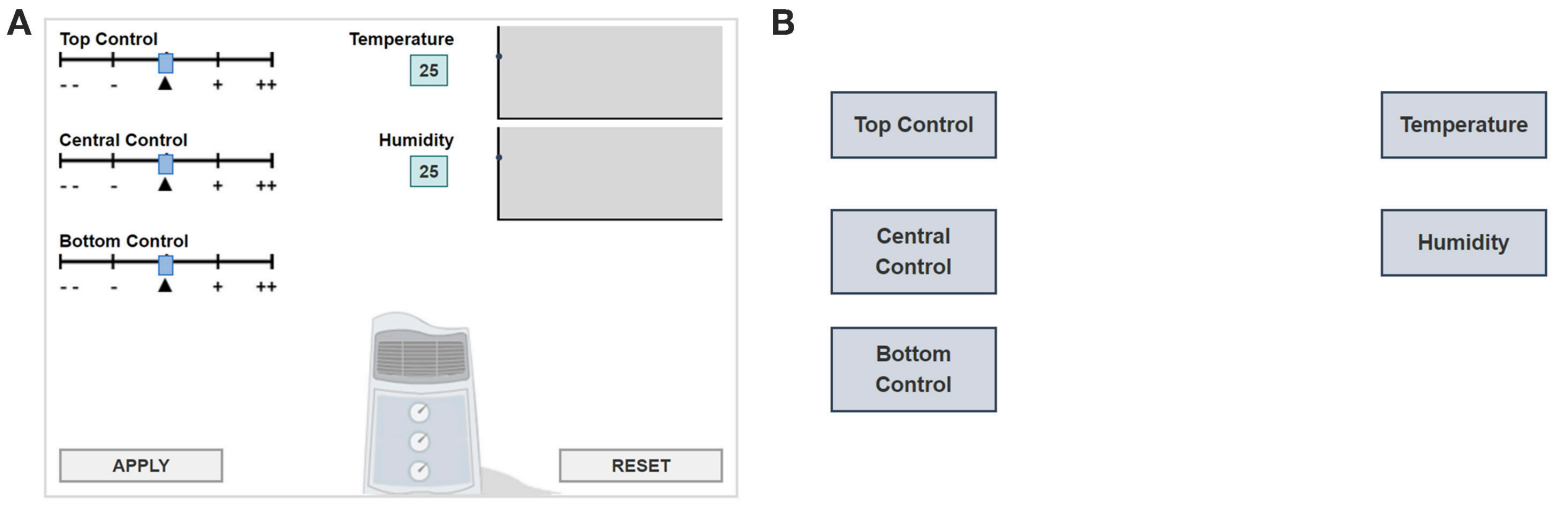
**(A)** Simulation environment of CC item. **(B)** Answer diagram of CC item.

In this item, students are instructed to manipulate the panel (i.e., to move the top, central, and bottom control sliders; left side of Figure 1A) and to answer how the input variables (control sliders) are related to the output variables (temperature and humidity). Specifically, the initial position of each control slider is indicated by a triangle “▴.” The students can change the top, central and bottom controls on the left of Figure 1 by using the sliders. By clicking “APPLY,” they will see the corresponding changes in temperature and humidity. After exploration, the students are asked to draw lines in a diagram (Figure 1B) to answer what each slider controls. The item is considered correctly answered if the diagram is correctly completed. The problem-solving process for this item is that the students must experiment to determine which controls have an impact on temperature and which on humidity, and then represent the causal relations by drawing arrows between the three inputs (top, central, and bottom control sliders) and the two outputs (temperature and humidity).

**Figure 2:**
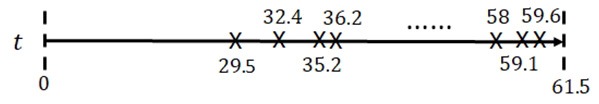
Visualization of the structure of process data from CC item in PISA 2012.

PISA 2012 collected students' problem-solving process data in computer log files, in the form of a sequence of time-stamped events. We illustrate the structure of data in Table 1 and Figure 2, where Table 1 tabulates a sequence of time-stamped events from a student and Figure 2 visualizes the corresponding event time points on a time line. According to the data, 14 events were recorded between time 0 (start) and 61.5 s (success). The first event happened at 29.5 s that was clicking “APPLY” after the top, central, and bottom controls were set at 2, 0, and 0, respectively. A sequence of actions followed the first event and finally at 58, 59.1, and 59.6 s, a final answer was correctly given using the diagram. It is worth clarifying that this log file does not collect all the interactions between a student and the simulated system. That is, the status of the control sliders is only recorded in the log file, when the “APPLY” button is clicked.

**Table 1:** An example of computer log file data from CC item in PISA 2012.

**Time**	**Event**
0	Start.
29.5	Set top, central, and bottom controls at 2, 0, and 0, respectively, and click APPLY.
32.4	Set top, central, and bottom controls at 0, 0, and 0, respectively, and click APPLY.
35.2	Click RESET.
36.2	Set all three controls at 0, and click APPLY.
⋮	⋮
58	Connecting ”top control” with ”temperature.”
59.1	Connecting ”central control” with ”humidity.”
59.6	Connecting ”bottom control” with ”humidity.”
61.5	Success.

The process data for solving a CPS item typically have two components, knowledge acquisition and knowledge application, respectively. This CC item mainly focuses the former, which includes learning the causal relationships between the inputs and the outputs and representing such relationships by drawing the diagram. Since data on representing the causal relationship is relatively straightforward, in the rest of the paper, we focus on the process data related to knowledge acquisition and only refer a student's problem-solving process to his/her process of exploring the air conditioner, excluding the actions involving the answer diagram.

Intuitively, students' problem-solving processes contain information about their complex problem-solving ability, whether in the context of the CC item or in a more general sense of dealing with complex tasks in practice. However, it remains a challenge to extract meaningful information from their process data, due to the complex data structure. In particular, the occurrences of events are heterogeneous (i.e., different people can have very different event histories) and unstructured (i.e., there is little restriction on the order and time of the occurrences). Different students tend to have different problem-solving trajectories, with different actions taken at different time points. Consequently, time series models, which are standard statistical tools for analyzing dynamic systems, are not suitable here.

### 2.2. Research Questions

We focus on two specific research questions. Consider an individual solving a complex problem. Given that the individual has spent *t* units of time and has not yet completed the task, we would like to ask the following two questions based on the information at time *t*: How much additional time does the individual need? And will the individual succeed or fail upon the time of task completion?

Suppose we index the individual by *i* and let *T*_*i*_ be the total time of task completion and *Y*_*i*_ be the final outcome. Moreover, we denote $H_{i}\left(t\right)={\left(h_{i1}\left(t\right),...,h_{ip}\left(t\right)\right)}^{⊤}$ as a *p*-vector function of time *t*, summarizing the event history of individual *i* from the beginning of task to time *t*. Each component of **H**_*i*_(*t*) is a feature constructed from the event history up to time *t*. Taking the above CC item as an example, components of **H**_*i*_(*t*) may be, the number of actions a student has taken, whether all three control sliders have been explored, the frequency of using the reset button, etc., up to time *t*. We refer to **H**_*i*_(*t*) as the event history process of individual *i*. The dimension *p* may be high, depending on the complexity of the log file.

With the above notation, the two questions become to simultaneously predict *T*_*i*_ and *Y*_*i*_ based on **H**_*i*_(*t*). Throughout this paper, we focus on the analysis of data from a single CPS item. Extensions of the current framework to multiple-item analysis are discussed in Section 5.

## 3. Proposed Method

### 3.1. A Regression Model

We now propose a regression model to answer the two questions raised in Section 2.2. We specify the marginal conditional models of *Y*_*i*_ and *T*_*i*_ given **H**_*i*_(*t*) and *T*_*i*_ > *t*, respectively. Specifically, we assume

(1) $$P\left(Y_{i}=1|H_{i}\left(t\right),T_{i}>t\right)=\Phi \left(b_{11}h_{i1}\left(t\right)+\cdots +b_{1p}h_{ip}\left(t\right)\right),$$

(2) $$E(\log(T_{i}-t)|H_{i}(t),T_{i}>t)=b_{21}h_{i1}(t)+\cdots +b_{2p}h_{ip}(t),$$

and

(3) $$Var(\log(T_{i}-t)|H_{i}(t),T_{i}>t)=\sigma^{2},$$

where Φ is the cumulative distribution function of a standard normal distribution. That is, *Y*_*i*_ is assumed to marginally follow a probit regression model. In addition, only the conditional mean and variance are assumed for log(*T*_*i*_−*t*). Our model parameters include the regression coefficients *B* = (_*b*_*jk*_)2 × *p*_ and conditional variance σ^2^. Based on the above model specification, a pseudo-likelihood function will be devived in Section 3.3 for parameter estimation.

Although only marginal models are specified, we point out that the model specifications (1) through (3) impose quite strong assumptions. As a result, the model may not most closely approximate the data-generating process and thus a bias is likely to exist. On the other hand, however, it is a working model that leads to reasonable prediction and can be used as a benchmark model for this prediction problem in future investigations.

We further remark that the conditional variance of log(*T*_*i*_−*t*) is time-invariant under the current specification, which can be further relaxed to be time-dependent. In addition, the regression model for response time is closely related to the log-normal model for response time analysis in psychometrics (e.g., van der Linden, 2007). The major difference is that the proposed model is not a measurement model disentangling item and person effects on *T*_*i*_ and *Y*_*i*_.

### 3.2. Prediction

Under the model in Section 3.1, given the event history, we predict the final outcome based on the success probability Φ(*b*_11_*h*_*i*1_(*t*) + ⋯ + *b*_1*p*_*h*_*ip*_(*t*)). In addition, based on the conditional mean of log(*T*_*i*_ − *t*), we predict the total time at time *t* by *t* + exp(*b*_21_*h*_*i*1_(*t*) + ⋯ + *b*_2*p*_*h*_*ip*_(*t*)). Given estimates of *B* from training data, we can predict the problem-solving duration and final outcome at any *t* for an individual in the testing sample, throughout his/her entire problem-solving process.

### 3.3. Parameter Estimation

It remains to estimate the model parameters based on a training dataset. Let our data be (τ_*i*_, *y*_*i*_) and {**H**_*i*_(*t*):*t* ≥ 0}, *i* = 1, …, *N*, where τ_*i*_ and *y*_*i*_ are realizations of *T*_*i*_ and *Y*_*i*_, and {**H**_*i*_(*t*): *t* ≥ 0} is the entire event history.

We develop estimating equations based on a pseudo likelihood function. Specifically, the conditional distribution of *Y*_*i*_ given **H**_*i*_(*t*) and *T*_*i*_ > *t* can be written as

$$f_{1}(y|H_{i}(t),\tau >t;\;b_{1})=\Phi {\left(b_{1}^{⊤}H_{i}(t)\right)}^{y}{\left(1-\Phi (b_{1}^{⊤}H_{i}(t))\right)}^{1-y},$$

where $b_{2}={\left(b_{11},...,b_{1p}\right)}^{⊤}$. In addition, using the log-normal model as a working model for *T*_*i*_−*t*, the corresponding conditional distribution of *T*_*i*_ can be written as

(4) $$\begin{array}{l} f_{2}(\tau |H_{i}(t),\tau >t;b_{2},\sigma )=\frac{1}{(\tau -t)\sigma \sqrt{2\pi }}\\ \;\exp\left(-\frac{{\left(\log(\tau -t)-(b_{2}^{⊤}H_{i}(t))\right)}^{2}}{2\sigma^{2}}\right), \end{array}$$

where $b_{2}={\left(b_{21},...,b_{2p}\right)}^{⊤}$. The pseudo-likelihood is then written as

(5) $$L(B,\sigma )=\prod_{i=1}^{N}\prod_{j=1}^{J}(f_{1}(y_{i}|H_{i}(t_{j}),\tau_{i}>t_{j};\;b_{1})f_{2}(\tau_{i}|H_{i}(t_{j}),\tau_{i}>t_{j};\;b_{2},\sigma ){)}^{1_{\left\{\tau_{i}>t_{j}\right\}}},$$

where *t*_1_, …, *t*_*J*_ are *J* pre-specified grid points that spread out over the entire time spectrum. The choice of the grid points will be discussed in the sequel. By specifying the pseudo-likelihood based on the sequence of time points, the prediction at different time is taken into accounting in the estimation. We estimate the model parameters by maximizing the pseudo-likelihood function *L*(*B*, σ).

In fact, (5) can be factorized into

$$L(B,\sigma )=L_{1}(b_{1})L_{2}(b_{2},\sigma ),$$

where

(6) $$L_{1}(b_{1})=\prod_{i=1}^{N}\prod_{j=1}^{J}(f_{1}(y_{i}|H_{i}(t_{j}),\tau_{i}>t_{j};\;b_{1}){)}^{1_{\left\{\tau_{i}>t_{j}\right\}}},$$

and

(7) $$L_{2}(b_{2},\sigma )=\prod_{i=1}^{N}\prod_{j=1}^{J}(f_{2}(\tau_{i}|H_{i}(t_{j}),\tau_{i}>t_{j};\;b_{2},\sigma ){)}^{1_{\left\{\tau_{i}>t_{j}\right\}}}.$$

Therefore, **b**_1_ is estimated by maximizing *L*_1_(**b**_1_), which takes the form of a likelihood function for probit regression. Similarly, **b**_2_ and σ are estimated by maximizing *L*_2_(**b**_2_, σ), which is equivalent to solving the following estimation equations,

(8) $$\sum_{i=1}^{N}\sum_{j=1}^{J}1_{\left\{\tau_{i}>t_{j}\right\}}(\log(\tau_{i}-t_{j})-\;b_{2}^{⊤}H_{i}(t_{j}))h_{ik}(t_{j})=0,k=1,...,p,$$

and

(9) $$\sum_{i=1}^{N}\sum_{j=1}^{J}1_{\left\{\tau_{i}>t_{j}\right\}}(\sigma^{2}-{\left(\log(\tau_{i}-t_{j})-\;b_{2}^{⊤}H_{i}(t_{j})\right)}^{2})=0.$$

The estimating equations (8) and (9) can also be derived directly based on the conditional mean and variance specification of log(*T*_*i*_−*t*). Solving these equations is equivalent to solving a linear regression problem, and thus is computationally easy.

### 3.4. Some Remarks

We provide a few remarks. First, choosing suitable features into **H**_*i*_(*t*) is important. The inclusion of suitable features not only improves the prediction accuracy, but also facilitates the exploratory analysis and interpretation of how behavioral patterns affect CPS result. If substantive knowledge about a CPS task is available from cognition theory, one may choose features that indicate different strategies toward solving the task. Otherwise, a data-driven approach may be taken. That is, one may select a model from a candidate list based on certain cross-validation criteria, where, if possible, all reasonable features should be consider as candidates. Even when a set of features has been suggested by cognition theory, one can still take the data-driven approach to find additional features, which may lead to new findings.

Second, one possible extension of the proposed model is to allow the regression coefficients to be a function of time *t*, whereas they are independent of time under the current model. In that case, the regression coefficients become functions of time, *b*_*jk*_(*t*). The current model can be regarded as a special case of this more general model. In particular, if *b*_*jk*_(*t*) has high variation along time in the best predictive model, then simply applying the current model may yield a high bias. Specifically, in the current estimation procedure, a larger grid point tends to have a smaller sample size and thus contributes less to the pseudo-likelihood function. As a result, a larger bias may occur in the prediction at a larger time point. However, the estimation of the time-dependent coefficient is non-trivial. In particular, constraints should be imposed on the functional form of *b*_*jk*_(*t*) to ensure a certain level of smoothness over time. As a result, *b*_*jk*_(*t*) can be accurately estimated using information from a finite number of time points. Otherwise, without any smoothness assumptions, to predict at any time during one's problem-solving process, there are an infinite number of parameters to estimate. Moreover, when a regression coefficient is time-dependent, its interpretation becomes more difficult, especially if the sign changes over time.

Third, we remark on the selection of grid points in the estimation procedure. Our model is specified in a continuous time domain that supports prediction at any time point in a continuum during an individual's problem-solving process. The use of discretized grid points is a way to approximate the continuous-time system, so that estimation equations can be written down. In practice, we suggest to place the grid points based on the quantiles of the empirical distribution of duration based on the training set. See the analysis in Section 4 for an illustration. The number of grid points may be further selected by cross validation. We also point out that prediction can be made at any time point on the continuum, not limited to the grid points for parameter estimation.

## 4. An Example from PISA 2012

### 4.1. Background

**Figure 3:**
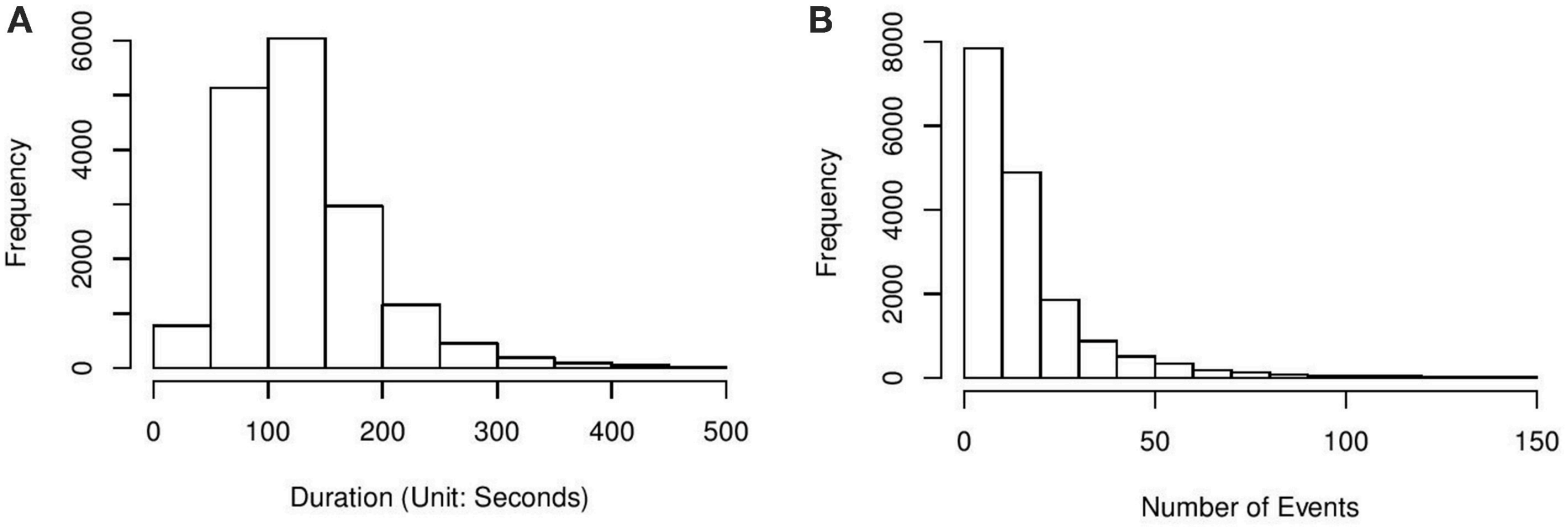
**(A)** Histogram of problem-solving duration of the CC item. **(B)** Histogram of the number of actions for solving the CC item.

In what follows, we illustrate the proposed method via an application to the above CC item^2^. This item was also analyzed in Greiff et al. (2015) and Xu et al. (2018). The dataset was cleaned from the entire released dataset of PISA 2012. It contains 16,872 15-year-old students' problem-solving processes, where the students were from 42 countries and economies. Among these students, 54.5% answered correctly. On average, each student took 129.9 s and 17 actions solving the problem. Histograms of the students' problem-solving duration and number of actions are presented in Figure 3.

### 4.2. Analyses

The entire dataset was randomly split into training and testing sets, where the training set contains data from 13,498 students and the testing set contains data from 3,374 students. A predictive model was built solely based on the training set and then its performance was evaluated based on the testing set. We used *J* = 9 grid points for the parameter estimation, with *t*_1_ through *t*_9_ specified to be 64, 81, 94, 106, 118, 132, 149, 170, and 208 s, respectively, which are the 10% through 90% quantiles of the empirical distribution of duration. As discussed earlier, the number of grid points and their locations may be further engineered by cross validation.

#### 4.2.1. Model Selection

We first build a model based on the training data, using a data-driven stepwise forward selection procedure. In each step, we add one feature into **H**_*i*_(*t*) that leads to maximum increase in a cross-validated log-pseudo-likelihood, which is calculated based on a five-fold cross validation. We stop adding features into **H**_*i*_(*t*) when the cross-validated log-pseudo-likelihood stops increasing. The order in which the features are added may serve as a measure of their contribution to predicting the CPS duration and final outcome.

The candidate features being considered for model selection are listed in Table 2. These candidate features were chosen to reflect students' CPS behavioral patterns from different aspects. In what follows, we discuss some of them. For example, the feature *I*_*i*_(*t*) indicates whether or not all three control sliders have been explored by simple actions (i.e., moving one control slider at a time) up to time *t*. That is, *I*_*i*_(*t*) = 1 means that the vary-one-thing-at-a-time (VOTAT) strategy (Greiff et al., 2015) has been taken. According to the design of the CC item, the VOTAT strategy is expected to be a strong predictor of task success. In addition, the feature *N*_*i*_(*t*)/*t* records a student's average number of actions per unit time. It may serve as a measure of the student's speed of taking actions. In experimental psychology, response time or equivalently speed has been a central source for inferences about the organization and structure of cognitive processes (e.g., Luce, 1986), and in educational psychology, joint analysis of speed and accuracy of item response has also received much attention in recent years (e.g., van der Linden, 2007; Klein Entink et al., 2009). However, little is known about the role of speed in CPS tasks. The current analysis may provide some initial result on the relation between a student's speed and his/her CPS performance. Moreover, the features defined by the repeating of previously taken actions may reflect students' need of verifying the derived hypothesis on the relation based on the previous action or may be related to students' attention if the same actions are repeated many times. We also include 1, *t, t*^2^, and *t*^3^ in **H**_*i*_(*t*) as the initial set of features to capture the time effect. For simplicity, country information is not taken into account in the current analysis.

**Table 2:** The list of candidate features to be incorporated into the model.

	**Feature**	**Explanation**
1.	*N*_*i*_(*t*)	Number of actions taken up to time *t*.
2.	*N*_*i*_(*t*)/*t*	Frequency of actions up to time *t*.
3.	1_{_*N*__*i*_(*t*)>0}_	Indicator of whether an action has been taken before time *t*.
4.	*S*_*i*_(*t*)	Number of simple actions (i.e., moving one control slider at a time)
		taken up to time *t*.
5.	*S*_*i*_(*t*)/*t*	Frequency of simple actions up to time *t*.
6.	1_{_*S*__*i*_(*t*)>0}_	Indicator of whether a simple action has been taken before time *t*.
7.	*I*_*i*_(*t*)	An indicator function, *I*_*i*_(*t*) = 1 if all three control sliders
		have been explored via simple actions up to time *t* and *I*_*i*_(*t*) = 0, otherwise.
8.	*R*_*i*_(*t*)	Number of RESET used up to time *t*.
9.	*R*_*i*_(*t*)/*t*	Frequency of RESET up to time *t*.
10.	1_{_*R*__*i*_(*t*)>0}_	Indicator of whether RESET has been used before time *t*.
11.	*RP*_*i*_(*t*)	Number of times that previously taken actions (excluding RESET)are repeated.
12.	*RP*_*i*_(*t*)/*t*	Frequency of repeating previously taken actions (excluding RESET).
13.	1_{*R*_*P*__*i*_(*t*)>0}_	Indicator of repeating previously taken actions (excluding RESET).

**Figure 4:**
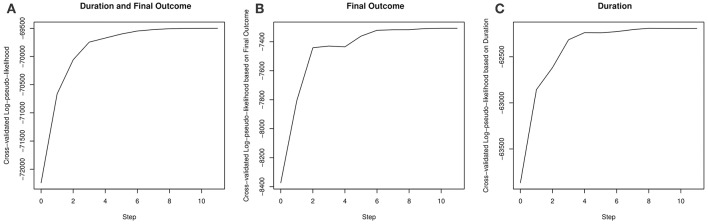
The increase in the cross-validated log-pseudo-likelihood based on a stepwise forward selection procedure. **(A–C)** plot the cross-validated log-pseudo-likelihood, corresponding to *L*(*B*, σ), *L*_1_(**b**_1_), *L*_2_(**b**_2_, σ), respectively.

Our results on model selection are summarized in Figure 4 and Table 3. The pseudo-likelihood stopped increasing after 11 steps, resulting a final model with 15 components in **H**_*i*_(*t*). As we can see from Figure 4, the increase in the cross-validated log-pseudo-likelihood is mainly contributed by the inclusion of features in the first six steps, after which the increment is quite marginal. As we can see, the first, second, and sixth features entering into the model are all related to taking simple actions, a strategy known to be important to this task (e.g., Greiff et al., 2015). In particular, the first feature being selected is *I*_*i*_(*t*), which confirms the strong effect of the VOTAT strategy. In addition, the third and fourth features are both based on *N*_*i*_(*t*), the number of actions taken before time *t*. Roughly, the feature 1_{_*N*__*i*_(*t*)>0}_ reflects the initial planning behavior (Eichmann et al., 2019). Thus, this feature tends to measure students' speed of reading the instruction of the item. As discussed earlier, the feature *N*_*i*_(*t*)/*t* measures students' speed of taking actions. Finally, the fifth feature is related to the use of the RESET button.

**Table 3:** Results on model selection based on a stepwise forward selection procedure.

**Step**	**Var.add**	**Lik**	**Lik.out**	**Lik.dur**
0.	1, *t*, *t*^2^, *t*^3^	–72241.7	–63867.9	–8373.7
1.	*I*_*i*_(*t*)	–70663.0	–62856.1	–7806.9
2.	1_{_*S*__*i*_(*t*)>0}_	–70058.3	–62617.0	–7441.4
3.	1_{_*N*__*i*_(*t*)>0}_	–69744.9	–62315.2	–7429.7
4.	*N*_*i*_(*t*)/*t*	–69672.7	–62237.6	–7435.1
5.	1_{_*R*__*i*_(*t*)>0}_	–69601.3	–62239.9	–7361.4
6.	*S*_*i*_(*t*)/*t*	-69547.6	–62226.8	–7320.8
7.	*RP*_*i*_(*t*)/*t*	–69522.5	–62205.1	–7317.4
8.	1_{*R*_*P*__*i*_(*t*)>0}_	–69507.0	-62190.0	–7317.0
9.	*R*_*i*_(*t*)	–69500.8	–62191.9	–7308.9
10.	*N*_*i*_(*t*)	–69499.4	–62192.6	–7306.8
11.	*RP*_*i*_(*t*)	–69498.5	–62191.8	–7306.7

*The columns “Lik,” “Lik.out,” and “Lik.dur” give the value of the cross-validated log-pseudo-likelihood, corresponding to L(B, σ), L_1_(**b**_1_), L_2_(**b**_2_, σ), respectively*.

#### 4.2.2. Prediction Performance on Testing Set

We now look at the prediction performance of the above model on the testing set. The prediction performance was evaluated at a larger set of time points from 19 to 281 s. Instead of reporting based on the pseudo-likelihood function, we adopted two measures that are more straightforward. Specifically, we measured the prediction of final outcome by the Area Under the Curve (AUC) of the predicted Receiver Operating Characteristic (ROC) curve. The value of AUC is between 0 and 1. A larger AUC value indicates better prediction of the binary final outcome, with AUC = 1 indicating perfect prediction. In addition, at each time point *t*, we measured the prediction of duration based on the root mean squared error (RMSE), defined as

$$\sqrt{\frac{\sum_{i=N+1}^{N+n}1_{\left\{\tau_{i}>t\right\}}{\left(\tau_{i}-{\hat{\tau }}_{i}(t)\right)}^{2}}{\sum_{i=N+1}^{N+n}1_{\left\{\tau_{i}>t\right\}}}},$$

where τ_*i*_, *i* = *N* + 1, …, *N* + *n*, denotes the duration of students in the testing set, and ${\hat{\tau }}_{i}\left(t\right)$ denotes the prediction based on information up to time *t* according to the trained model.

**Figure 5:**
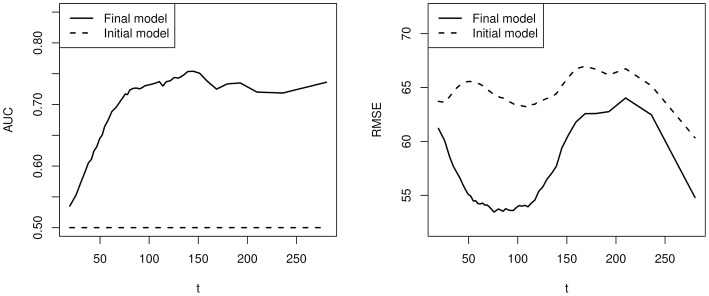
A comparison of prediction accuracy between the model selected by cross validation and a baseline model without using individual specific event history.

Results are presented in Figure 5, where the testing AUC and RMSE for the final outcome and duration are presented. In particular, results based on the model selected by cross validation (*p* = 15) and the initial model (*p* = 4, containing the initial covariates 1, *t*, *t*^2^, and *t*^3^) are compared. First, based on the selected model, the AUC is never above 0.8 and the RMSE is between 53 and 64 s, indicating a low signal-to-noise ratio. Second, the students' event history does improve the prediction of final outcome and duration upon the initial model. Specifically, since the initial model does not take into account the event history, it predicts the students with duration longer than *t* to have the same success probability. Consequently, the test AUC is 0.5 at each value of *t*, which is always worse than the performance of the selected model. Moreover, the selected model always outperforms the initial model in terms of the prediction of duration. Third, the AUC for the prediction of the final outcome is low when *t* is small. It keeps increasing as time goes on and fluctuates around 0.72 after about 120 s.

#### 4.2.3. Interpretation of Parameter Estimates

To gain more insights into how the event history affects the final outcome and duration, we further look at the results of parameter estimation. We focus on a model whose event history **H**_*i*_(*t*) includes the initial features and the top six features selected by cross validation. This model has similar prediction accuracy as the selected model according to the cross-validation result in Figure 4, but contains less features in the event history and thus is easier to interpret. Moreover, the parameter estimates under this model are close to those under the cross-validation selected model, and the signs of the regression coefficients remain the same.

The estimated regression coefficients are presented in Table 4. First, the first selected feature *I*_*i*_(*t*), which indicates whether all three control sliders have been explored via simple actions, has a positive regression coefficient on final outcome and a negative coefficient on duration. It means that, controlling the rest of the parameters, a student who has taken the VOTAT strategy tends to be more likely to give a correct answer and to complete in a shorter period of time. This confirms the strong effect of VOTAT strategy in solving the current task.

**Table 4:** Estimated regression coefficients for a model for which the event history process contains the initial features based on polynomials of *t* and the top six features selected by cross validation.

	**Feature**	${\hat{b}}_{1}$	${\hat{b}}_{2}$
1.	1	3.1 × 10^−1^	4.8
2.	*t*	−5.9 × 10^−3^	−2.7 × 10^−3^
3.	*t*^2^	3.1 × 10^−6^	−4.5 × 10^−7^
4.	*t*^3^	1.7 × 10^−8^	3.5 × 10^−8^
5.	*I*_*i*_(*t*)	5.2 × 10^−1^	−8.4 × 10^−1^
6.	1_{_*S*__*i*_(*t*)>0}_	6.8 × 10^−1^	−2.1 × 10^−1^
7.	1_{_*N*__*i*_(*t*)>0}_	−3.1 × 10^−1^	−6.6 × 10^−1^
8.	*N*_*i*_(*t*)/*t*	−1.1	−1.4
9.	1_{_*R*__*i*_(*t*)>0}_	3.7 × 10^−1^	3.8 × 10^−2^
10.	*S*_*i*_(*t*)/*t*	3.0	7.9 × 10^−1^

Second, besides *I*_*i*_(*t*), there are two features related to taking simple actions, 1_{_*S*__*i*_(*t*)>0}_ and *S*_*i*_(*t*)/*t*, which are the indicator of taking at least one simple action and the frequency of taking simple actions. Both features have positive regression coefficients on the final outcome, implying larger values of both features lead to a higher success rate. In addition, 1_{_*S*__*i*_(*t*)>0}_ has a negative coefficient on duration and *S*_*i*_(*t*)/*t* has a positive one. Under this estimated model, the overall simple action effect on duration is ${\hat{b}}_{25}I_{i}\left(t\right)+{\hat{b}}_{26}1_{\left\{S_{i}\left(t\right)>0\right\}}+{\hat{b}}_{2,10}S_{i}\left(t\right)/t$, which is negative for most students. It implies that, overall, taking simple actions leads to a shorter predicted duration. However, once all three types of simple actions have been taken, a higher frequency of taking simple actions leads to a weaker but sill negative simple action effect on the duration.

Third, as discussed earlier, 1_{_*N*__*i*_(*t*)>0}_ tends to measure the student's speed of reading the instruction of the task and *N*_*i*_(*t*)/*t* can be regarded as a measure of students' speed of taking actions. According to the estimated regression coefficients, the data suggest that a student who reads and acts faster tends to complete the task in a shorter period of time with a lower accuracy. Similar results have been seen in the literature of response time analysis in educational psychology (e.g., Klein Entink et al., 2009; Fox and Marianti, 2016; Zhan et al., 2018), where speed of item response was found to negatively correlated with accuracy. In particular, Zhan et al. (2018) found a moderate negative correlation between students' general mathematics ability and speed under a psychometric model for PISA 2012 computer-based mathematics data.

Finally, 1_{_*R*__*i*_(*t*)>0}_, the use of the RESET button, has positive regression coefficients on both final outcome and duration. It implies that the use of RESET button leads to a higher predicted success probability and a longer duration time, given the other features controlled. The connection between the use of the RESET button and the underlying cognitive process of complex problem solving, if it exists, still remains to be investigated.

## 5. Discussions

### 5.1. Summary

As an early step toward understanding individuals' complex problem-solving processes, we proposed an event history analysis method for the prediction of the duration and the final outcome of solving a complex problem based on process data. This approach is able to predict at any time *t* during an individual's problem-solving process, which may be useful in dynamic assessment/learning systems (e.g., in a game-based assessment system). An illustrative example is provided that is based on a CPS item from PISA 2012.

### 5.2. Inference, Prediction, and Interpretability

As articulated previously, this paper focuses on a prediction problem, rather than a statistical inference problem. Comparing with a prediction framework, statistical inference tends to draw stronger conclusions under stronger assumptions on the data generation mechanism. Unfortunately, due to the complexity of CPS process data, such assumptions are not only hardly satisfied, but also difficult to verify. On the other hand, a prediction framework requires less assumptions and thus is more suitable for exploratory analysis. As a price, the findings from the predictive framework are preliminary and can only be used to generate hypotheses for future studies.

It may be useful to provide uncertainty measures for the prediction performance and for the parameter estimates, where the former indicates the replicability of the prediction performance and the later reflects the stability of the prediction model. In particular, patterns from a prediction model with low replicability and low stability should not be overly interpreted. Such uncertainty measures may be obtained from cross validation and bootstrapping (see Chapter 7, Friedman et al., 2001).

It is also worth distinguishing prediction methods based on a simple model like the one proposed above and those based on black-box machine learning algorithms (e.g., random forest). Decisions based on black-box algorithms can be very difficult to understood by human and thus do not provide us insights about the data, even though they may have a high prediction accuracy. On the other hand, a simple model can be regarded as a data dimension reduction tool that extracts interpretable information from data, which may facilitate our understanding of complex problem solving.

### 5.3. Extending the Current Model

The proposed model can be extended along multiple directions. First, as discussed earlier, we may extend the model by allowing the regression coefficients *b*_*jk*_ to be time-dependent. In that case, nonparametric estimation methods (e.g., splines) need to be developed for parameter estimation. In fact, the idea of time-varying coefficients has been intensively investigated in the event history analysis literature (e.g., Fan et al., 1997). This extension will be useful if the effects of the features in **H**_*i*_(*t*) change substantially over time.

Second, when the dimension *p* of **H**_*i*_(*t*) is high, better interpretability and higher prediction power may be achieved by using Lasso-type sparse estimators (see e.g., Chapter 3 Friedman et al., 2001). These estimators perform simultaneous feature selection and regularization in order to enhance the prediction accuracy and interpretability.

Finally, outliers are likely to occur in the data due to the abnormal behavioral patterns of a small proportion of people. A better treatment of outliers will lead to better prediction performance. Thus, a more robust objective function will be developed for parameter estimation, by borrowing ideas from the literature of robust statistics (see e.g., Huber and Ronchetti, 2009).

### 5.4. Multiple-Task Analysis

The current analysis focuses on analyzing data from a single task. To study individuals' CPS ability, it may be of more interest to analyze multiple CPS tasks simultaneously and to investigate how an individual's process data from one or multiple tasks predict his/her performance on the other tasks. Generally speaking, one's CPS ability may be better measured by the information in the process data that is generalizable across a representative set of CPS tasks than only his/her final outcomes on these tasks. In this sense, this cross-task prediction problem is closely related to the measurement of CPS ability. This problem is also worth future investigation.

## Author Contributions

All authors listed have made a substantial, direct and intellectual contribution to the work, and approved it for publication.

### Conflict of Interest Statement

The authors declare that the research was conducted in the absence of any commercial or financial relationships that could be construed as a potential conflict of interest.

## Footnotes

^1^The item can be found on the OECD website (http://www.oecd.org/pisa/test-2012/testquestions/question3/)

^2^The log file data and code book for the CC item can be found online: http://www.oecd.org/pisa/pisaproducts/database-cbapisa2012.htm.

## References

[B1] Allison P. D. (2014). Event history analysis: Regression for longitudinal event data. London: Sage.

[B2] Danner D. Hagemann D. Schankin A. Hager M. Funke J. (2011). Beyond IQ: a latent state-trait analysis of general intelligence, dynamic decision making, and implicit learning. Intelligence 39, 323–334. 10.1016/j.intell.2011.06.004

[B3] Eichmann B. Goldhammer F. Greiff S. Pucite L. Naumann J. (2019). The role of planning in complex problem solving. Comput. Educ. 128, 1–12. 10.1016/j.compedu.2018.08.004

[B4] Fan J. Gijbels I. King M. (1997). Local likelihood and local partial likelihood in hazard regression. Anna. Statist. 25, 1661–1690. 10.1214/aos/1031594736

[B5] Fox J.-P. Marianti S. (2016). Joint modeling of ability and differential speed using responses and response times. Multivar. Behav. Res. 51, 540–553. 10.1080/00273171.2016.1171128 27269482

[B6] Friedman J. Hastie T. Tibshirani R. (2001). The Elements of Statistical Learning. New York, NY: Springer.

[B7] Greiff S. Wüstenberg S. Avvisati F. (2015). Computer-generated log-file analyses as a window into students' minds? A showcase study based on the PISA 2012 assessment of problem solving. Comput. Educ. 91, 92–105. 10.1016/j.compedu.2015.10.018

[B8] Greiff S. Wüstenberg S. Funke J. (2012). Dynamic problem solving: a new assessment perspective. Appl. Psychol. Measur. 36, 189–213. 10.1177/0146621612439620

[B9] Halpin P. F. De Boeck P. (2013). Modelling dyadic interaction with Hawkes processes. Psychometrika 78, 793–814. 10.1007/s11336-013-9329-1 24092489

[B10] Halpin P. F. von Davier A. A. Hao J. Liu L. (2017). Measuring student engagement during collaboration. J. Educ. Measur. 54, 70–84. 10.1111/jedm.12133

[B11] He Q. von Davier M. (2015). Identifying feature sequences from process data in problem-solving items with N-grams, in Quantitative Psychology Research, eds van der Ark L. Bolt D. Wang W. Douglas J. Wiberg M. (New York, NY: Springer), 173–190.

[B12] He Q. von Davier M. (2016). Analyzing process data from problem-solving items with n-grams: insights from a computer-based large-scale assessment, in Handbook of Research on Technology Tools for Real-World Skill Development, eds Rosen Y. Ferrara S. Mosharraf M. (Hershey, PA: IGI Global), 750–777.

[B13] Huber P. J. Ronchetti E. (2009). Robust Statistics. Hoboken, NJ: John Wiley & Sons.

[B14] Klein Entink R. H. Kuhn J.-T. Hornke L. F. Fox J.-P. (2009). Evaluating cognitive theory: A joint modeling approach using responses and response times. Psychol. Methods 14, 54–75. 10.1037/a0014877 19271848

[B15] Luce R. D. (1986). Response Times: Their Role in Inferring Elementary Mental Organization. New York, NY: Oxford University Press.

[B16] MacKay D. G. (1982). The problems of flexibility, fluency, and speed–accuracy trade-off in skilled behavior. Psychol. Rev. 89, 483–506. 10.1037/0033-295X.89.5.483

[B17] van der Linden W. J. (2007). A hierarchical framework for modeling speed and accuracy on test items. Psychometrika 72, 287–308. 10.1007/s11336-006-1478-z

[B18] Vista A. Care E. Awwal N. (2017). Visualising and examining sequential actions as behavioural paths that can be interpreted as markers of complex behaviours. Comput. Hum. Behav. 76, 656–671. 10.1016/j.chb.2017.01.027

[B19] Wüstenberg S. Greiff S. Funke J. (2012). Complex problem solving–More than reasoning? Intelligence 40, 1–14. 10.1016/j.intell.2011.11.003

[B20] Xu H. Fang G. Chen Y. Liu J. Ying Z. (2018). Latent class analysis of recurrent events in problem-solving items. Appl. Psychol. Measur. 42, 478–498. 10.1177/0146621617748325 30787489 PMC6373852

[B21] Yarkoni T. Westfall J. (2017). Choosing prediction over explanation in psychology: lessons from machine learning. Perspect. Psychol. Sci. 12, 1100–1122. 10.1177/1745691617693393 28841086 PMC6603289

[B22] Zhan P. Jiao H. Liao D. (2018). Cognitive diagnosis modelling incorporating item response times. Br. J. Math. Statist. Psychol. 71, 262–286. 10.1111/bmsp.12114 28872185

